# Editorial: The Major Discoveries of Cajal and His Disciples: Consolidated Milestones for the Neuroscience of the XXIst Century

**DOI:** 10.3389/fnana.2016.00085

**Published:** 2016-10-13

**Authors:** Fernando de Castro, Miguel A. Merchán

**Affiliations:** ^1^Grupo de Neurobiología del Desarrollo, Instituto Cajal-CSICMadrid, Spain; ^2^Instituto de Neurociencias de Castilla y León, Universidad de SalamancaSalamanca, Spain

**Keywords:** History of Neuroscience, History of Science, neuron theory, neuronism, Cajal, synapse, dendritic spine, glia

When Santiago Ramón y Cajal (1904; 1911) began to understand the fine structure of the nervous system in the last decades of the XIXth century, he was almost certainly alone in his conviction that his descriptions were scientific truths, many of which have outlived him and remain relevant. Simple histological staining, a monocular microscope, an unabated curiosity, patience and a special talent to represent his observations in sketches, and drawings, as well as a rich imaginative and open mind, all elements that together account for Cajal's success. His descriptions of the connectivity in the nervous system were compiled into an opus magna that was first published in 1904 (“Textura del sistema nervioso del hombre y los vertebrados,” in Spanish) and subsequently translated into French in 1911 (“Histologie du systeme nerveux”). As the decades have passed, one by one all his theories have been corroborated using modern techniques, and the main hypotheses that Cajal postulated have become universally recognized as biological laws: The neuron theory; the law of the dynamic polarization of the neuron and the principle of connectional specificity.

Attracted by Cajal's strong personality, a number of medical students and doctors joined him in his crusade to explore the nervous system, and the seeds planted by the many faceted savant proved to be truly fructiferous. As such, among the successes of his disciples: Francisco Tello described interesting aspects of the regeneration of peripheral nerves, which still prove to be useful to neuroscientists currently working in this field; Nicolás Achúcarro contributed significantly to the study of neuroglia and subsequently, microglia; Pío del Río-Hortega identified two of the four main types of nervous cell, the oligodendrocytes and microglia, and his proposed classification for CNS tumors remains valid today; Fernando de Castro was the first person to describe the existence of arterial chemoreceptors to control blood composition, and who place them in the carotid body; Rafael Lorente de Nó was a dominant figure in Neuroscience for decades after the IInd World War, first describing the columnar organization of the cerebral cortex, well before the confirmatory works of Mountcastle, Hubbel, and Wiesel. In addition, several of the less well recognized co-workers and disciples of Cajal made discoveries that have been consolidated and are considered scientific truths today, including his brother Pedro Ramón y Cajal, Domingo Sánchez, and the neurologist Rodríguez-Lafora.

As a whole, it is difficult to find a scientific school that has contributed in such a fundamental and varied way to our common cultural heritage as that of Cajal. Cajal's particular way of working involved sharing his enthusiasm with select group of promising and proven collaborators, and giving them freedom to carry out their studies. This format has been recognized and compared to other systems established by relevant contemporary scientists and scientific schools, although maybe no other has marked such significant milestones in Biomedical Sciences that remain relevant well into the XXIst century. The main purpose of this Special Research Topic is to remind us of all these examples of how successful scientific study may become when attention is paid to detail, and brilliant, imaginative and skilled scientists work with unwavering commitment despite receiving minimal support.

An analysis of the scientific legacy of Cajal and his disciples (collectively known as the Spanish Neurohistological School, or more colloquially, Cajal's School) is tantamount to studying the fundamentals of the organization and function of the nervous system. In this Research Topic, the main contributions of the Spanish Neurohistological School are discussed in depth and in the light of the current dogma in Neuroscience. In this sense we would like to emphasize that the *corpus doctrinae* upon which this Research Topic is founded constitutes a solid conceptual guide for future generations of neuroscientists. The contributions focus on the most relevant areas in which Cajal and his disciples influenced neuroscience today. In this issue, the outstanding and experienced panel of contributors offer new perspectives on Cajal's work and theories, allowing the reader to obtain many relevant new insights into how the brain and mind works.

Dr. Delgado-García's takes a view of Cajal's work centered on the basic principles of cortical and nuclear organization, as well as addressing the old and new meanings of neural plasticity. Many interesting questions and hypotheses appear in his contribution, following on from a deep conceptual incursion in the world of the mind of Cajal. Very interesting and original papers from Dr. Blanco and Drs. Rozo and Rodríguez Moreno, respectively, analyse the historical and international context of the period in which Cajal was active, drawing comparisons with another great contemporary scientist, Ivan Pavlov. Addressing the parallels between their Nobel prizes in relation to Darwin's theory of Evolution, the reasons why Cajal extensively analyzed comparative anatomy become clear in this article, a search for the principles of organization and specificity of the connections in the nervous system. A complete overview of the influence of Darwin's ideas, and of the contribution of Cajal's theories on neuronal network plasticity and learning is presented by Ferreira et al. This article can be considered as one of the most relevant contributions to understand the true added value of Cajal's ideas to modern Neuroscience.

The paper dealing with the discovery of growth cones in the developing and lessioned nervous system by Drs. Tamaríz and Varela-Echevarría is a key piece of work in this issue, critical to fully understanding how Cajal derives his hypothesize about the dynamic reorganization of the nervous system. This paper presents a well-balanced combination of historical and recent data on growth cones and axon guidance, allowing the reader to obtain a clear idea about the chemotropic postulate in the development and regeneration of the nervous system. Like growth cones, dendritic spines were for many of Cajal's contemporary scientists simply artifacts of staining and/or fixation. In this issue a paper from Dr. Yuste comprehensively describes how Cajal concluded that dendritic spines are not only active structures on neurons but, more importantly, active elements relevant for central nervous system plasticity and connectivity. These two papers highlight the impact today of two of Cajal's more relevant milestones (growth cones and spines) on our understanding of the nervous system's capacity to change and adapt.

The Cajal-Retzius cells that lie in layer I of the brain cortex represent one of Cajal's more enigmatic findings, and these cells have been analyzed in depth in this Research Topic from three converging points of view. Dr. Marín-Padilla, one of Cajal's most enthusiastic followers, contributes a manuscript that adopts the perspective of Cajal as a pioneer in analyzing this type of neuron in depth, and of an expert in applying silver techniques to human brain sections. This paper is unique in this Research Topic, as far from simply extending a bridge from the historical to the modern perspective, it offers us insight to understand Cajal's way of thinking. Both, the historical peripeteia that the discovery of Cajal-Retzius cells represents and the current perspective of the role of these neurons in the developing cortex are analyzed extensively in two important papers by Drs. Gil et al. and by Drs. Martínez-Cerdeño and Noctor.

As an example of Cajal's descriptions of the sensory system, the well written article about the olfactory system by Drs. Figueres-Oñate et al. deserves particular mention. This article is peppered with beautiful images that are analyzed meticulously, and it presents Cajal's complete anatomical descriptions of olfactory bulb cells and the connectivity of central pathways. A very interesting paper by Dr. Navarrete and Araque demonstrates that our current ideas about the active role of astrocytes in regulating neuronal activity were implicit in several pieces of Cajal's work and that of some of his disciples. Their splendid analysis highlights once more the current validity of the hypotheses postulated by Cajal.

The most outstanding contributions to neuroscience of Pío del Río-Hortega can be found in a set of three papers in this Research Topic. A masterful overview of his contribution to the discovery of microglial cells, and that of his mentor Nicolás Achúcarro, together with a novel insight into the future of functional neuroanatomy is presented by Drs. Tremblay et al. A mini-review by Drs. Pérez-Cerdá et al. also includes a summarized sketch of Río-Hortega's biography and the basis of the silver carbonate method that he developed for glial cell staining. In addition, their article includes a very interesting comparison of Río-Hortega's classification of oligodendrocytes with that currently accepted for glial subtypes. Finally, by focusing on the role of the Spanish Neurohistological School's contribution in pathology, Dr. Ramón y Cajal Agüeras offers a very interesting view about del Río-Hortega's pioneer analysis and classification of brain tumors.

The figure of Rafael Lorente de Nó, one of Cajal's disciples who had the greatest impact on modern Neurophysiology, is carefully analyzed and discussed in a paper written by Dr. Larriva-Sahd. This paper thoroughly describes Lorente's concept of the cortical columnar organization of the brain and of input segregation at the dendritic trees of pyramidal cells. It also addresses Lorente's main general theories, such as the neural basis of the unidirectional chain of neurons in reflexes, as well as the creative and outstanding functional models of subthreshold synaptic stimulation or temporo-spatial decoding in neuronal networks.

Finally, Dr. de Castro undertakes to explain the relevance of his grandfather, providing an extensive description, along with a beautiful collection of images from his personal historical files, he successfully outlines the scientific stature of Fernando de Castro and his contribution to our understanding of the peripheral nervous system, as well as his repercussions on current Neuroscience. Similarly, a magisterial analysis of Fernando de Castro's work on chemo- and baroreceptors is included by Dr Constancio Gonzalez et al.^1^ which definitively shows de Castro's central role in establishing the basis of the nervous system's regulation of blood pressure.

Unfortunately, we were unable to find authors to offer a suitable contribution on maybe the first of Cajal's disciples, Jorge Francisco Tello, even though the regeneration of the nervous system is currently is field of significant interest. Similarly, no-one was found to contribute a chapter on Gonzalo R. Lafora, the renowned neuropathologist that described progressive myoclonic epilepsy or Lafora's disease. This was a pity but time constraints impeded us from covering these topics.

To close the Special Research Topic, there is an eloquent epilog by Drs. Lerma and De Carlos that focuses on the dimension of Cajal's work, not only in the field of Neuroscience but also, as a leader in promoting modern Spanish science. This is a very well written historical sketch that allows the readers to obtain a more complete understanding of the wider dimension of Cajal and of his ability to train brilliant students and collaborators.

In conjunction, we think that this Research Topic represents an outstanding work on the History of Neuroscience and perhaps, one of the most complete analytical discussion of Cajal's life and legacy, and that of the Spanish Neurohistological School, published in English to date^2^. The subject matter well merits the effort that has been made, and we feel we have achieved our main objective: To show how pioneering, vast and valid were the discoveries of this group of scientists, and how they influenced, and continue to influence the evolution of Neuroscience as a discipline even today.

We would like to deeply acknowledge all contributors to this Research Topic for their effort and excellent papers.

## Author contributions

MM and FC have co-written this editorial.

## Funding

Our current research activities are financed with grants from the Spanish Ministerio de Economía y Competitividad-MINECO (SAF2012-40023, RD12-0032-12) and CSIC (2015201023) to FC.

### Conflict of interest statement

The authors declare that the research was conducted in the absence of any commercial or financial relationships that could be construed as a potential conflict of interest.

## Footnotes

^1^Unfortunately, the great Professor of Physiology and our beloved friend Constancio González could not view this Special Research Topic completed because he died at the begining of the summer 2015. His was one of the first contributions received for this Special Research Topic.

^2^In Spanish, we should remark that postumously published by Fernando de Castro, the last direct disciple of Santiago Ramón y Cajal (de Castro, 1981).
